# Supplementary material for: An evidence-based approach to artificial intelligence education for medical students: A systematic review
Source: PLOS Digit Health. 2023 Nov 27;2(11):e0000255. doi: 10.1371/journal.pdig.0000255 (PMC10681314; doi:10.1371/journal.pdig.0000255)
Supplement: S1 Table — The NOS criteria is comprised of three categories: selection, comparability, and outcome. (DOCX) [file pdig.0000255.s001.docx]

**S1 Table. A table that shows the NOS criteria and scoring for all the survey type studies included. The NOS criteria was composed of three categories: selection, comparability, and outcome.**

| Title | Selection | Comparability | Outcome (Cohort, Cross-sectional) |
| --- | --- | --- | --- |
| The impact of artificial intelligence on clinical education: perceptions of postgraduate trainee doctors in London (UK) and recommendations for trainers [7] | 3 | 1 | 1 |
| Health Care Students’ Perspectives on Artificial Intelligence: Countrywide Survey in Canada [8] | 3 | 1 | 1 |
| Medical artificial intelligence readiness scale for medical students (MAIRS-MS) – development, validity and reliability study [17] | 3 | 2 | 1 |
| Influence of Artificial Intelligence on Canadian Medical Students' Preference for Radiology Specialty: A National Survey Study [18] | 3 | 1 | 1 |
| Medical students' attitude towards artificial intelligence:  a multicentre survey [19] | 2 | 1 | 1 |
| Attitudes and perceptions of UK medical students towards artificial intelligence and radiology: a multicentre survey [20] | 3 | 1 | 2 |
| Medical Student Perspectives on the Impact of Artificial Intelligence on the Practice of Medicine [21] | 3 | 1 | 1 |
| Impact of artificial intelligence on US medical students' choice of radiology [22] | 3 | 1 | 2 |
| Radiology Community Attitude in Saudi Arabia about the Applications of Artificial Intelligence in Radiology [23] | 2 | 1 | 2 |
| Interventional radiology and artificial intelligence in radiology: Is it time to enhance the vision of our medical students? [24] | 3 | 1 | 1 |
| The Approaches and Expectations of the Health Sciences Students Towards Artificial Intelligence [25] | 2 | 1 | 1 |
| Artificial intelligence and medical education: A global mixed-methods study of medical students’ perspectives [26] | 3 | 1 | 1 |
| Medical Students’ Perceptions towards Digitization and Artificial Intelligence: A Mixed-Methods Study [27] | 3 | 1 | 1 |
| Machine learning in medical education: a survey of the experiences and opinions of medical students in Ireland [28] | 2 | 1 | 1 |
| A survey on the future of radiology among radiologists, medical students and surgeons: Students and surgeons tend to be more skeptical about artificial intelligence and radiologists may fear that other disciplines take over [29] | 3 | 1 | 2 |
| Are We Ready to Integrate Artificial Intelligence Literacy into Medical School Curriculum: Students and Faculty Survey [30] | 3 | 1 | 1 |
| Artificial Intelligence and the Future of Primary Care: Exploratory Qualitative Study of UK General Practitioners’ Views [31] | 3 | 1 | 1 |
| Computerization and the future of primary care: A survey of general practitioners in the UK [32] | 3 | 1 | 1 |
| US primary care in 2029: A Delphi survey on the impact of machine learning [33] | 3 | 0 | 1 |
| Medical student knowledge and critical appraisal of machine learning: a multicentre international cross-sectional study [34] | 3 | 1 | 1 |
| Differences in Knowledge and Perspectives on the Usage of Artificial Intelligence Among Doctors and Medical Students of a Developing Country: A Cross-Sectional Study [35] | 2 | 2 | 2 |
| Mapping the Access of Future Doctors to Health Information Technologies Training in the European Union: Cross-Sectional Descriptive Study [36] | 2 | 1 | 2 |
| Artificial Intelligence in Primary Health Care: Perceptions, Issues, and Challenges [45] | 2 | 2 | 1 |
