# Supplementary material for: An evidence-based approach to artificial intelligence education for medical students: A systematic review
Source: PLOS Digit Health. 2023 Nov 27;2(11):e0000255. doi: 10.1371/journal.pdig.0000255 (PMC10681314; doi:10.1371/journal.pdig.0000255)
Supplement: S2 Table — (DOCX) [file pdig.0000255.s002.docx]

**S2 Table. A table that shows the ROBIS criteria and scoring for all of the review studies included. Phase 2 identifies any concerns with the review process and phase 3 is an overall assessment of the risk of bias.**

| **Title** | **Phase 2** | | | | **Phase 3** |
| --- | --- | --- | --- | --- | --- |
|  | 1.Study Eligibility | 2.Identification and selection of studies | 3.Data collection and study appraisal | 4.Synthesis and findings | Risk of bias in review |
| Artificial Intelligence in Undergraduate Medical Education: A Scoping Review [12] | Low Risk | Low Risk | High Risk | Low Risk | Low Risk |
| Artificial Intelligence Education Programs for Health Care Professionals: Scoping Review [13] | Low Risk | Low Risk | Unclear | Low Risk | Low Risk |
| Stakeholders’ perspectives on the future of artificial intelligence in radiology: a scoping review [37] | Low Risk | Low Risk | Unclear | Low Risk | Low Risk |
| Applications and Challenges of Implementing Artificial Intelligence in Medical Education: Integrative Review [38] | Low Risk | High Risk | High Risk | Low Risk | Unclear |
| Artificial Intelligence Education and Tools for Medical and Health Informatics Students: Systematic Review [39] | Low Risk | Low Risk | Unclear | Low Risk | Low Risk |
| Medical education trends for future physicians in the era of advanced technology and artificial intelligence: an integrative review [40] | Low Risk | Low Risk | Low Risk | Low Risk | Low Risk |
| The Recent Progress and Applications of Digital Technologies in Healthcare: A Review [41] | High Risk | High Risk | High Risk | High Risk | High Risk |
| Digital health competencies in medical school education: a scoping review and Delphi method [42] | High Risk | High Risk | High Risk | Low Risk | High Risk |
| Digital Health Training Programs for Medical Students: Scoping Review [43] | Low Risk | Low Risk | Unclear | Low Risk | Low Risk |
| Systematic Review of Radiologist and Medical Student Attitudes on the Role and Impact of AI in Radiology [44] | Low Risk | High Risk | High Risk | High Risk | High Risk |
| Educating Future Physicians in Artificial Intelligence (AI): An Integrative Review and Proposed Changes [46] | Low Risk | Low Risk | High Risk | Low Risk | Low Risk |
