# Supplementary material for: An evidence-based approach to artificial intelligence education for medical students: A systematic review
Source: PLOS Digit Health. 2023 Nov 27;2(11):e0000255. doi: 10.1371/journal.pdig.0000255 (PMC10681314; doi:10.1371/journal.pdig.0000255)
Supplement: Attachment — Submitted filename: Response to Reviewers.docx [file pdig.0000255.s004.docx]

**Author Responses Document**

Manuscript Title: Artificial Intelligence Education for Medical Students: A Systematic Review

Authors: Nikola Pupic, Aryan Ghaffarizadeh, Ricky Hu, Rohit Singla, Anna Karwowska, Kathryn Darras, and Bruce Forster

We thank the reviewers for their insightful comments. The reviewers appreciate the importance of this paper in the emerging field of AI in medicine and were generally satisfied with the scope of the paper. We appreciate the feedback, in particular identifying the sections where clarifying information is necessary to help the readers better understand the importance of the subject. We have addressed all the comments below and also have indicated the changes made following the feedback of the reviewers. We have also included a copy of the manuscript with the tracked changes as well as a clean copy.

**Reviewer #1 (Remarks to the Author):**

**1. Reviewer Comment:** This systematic review attempts to identify emerging themes in incorporating artificial intelligence literacy in medical education. The authors do a nice job of synthesizing their findings into relevant, albeit broad, learning objectives for undergraduate medical curricula.

The study title is too broad and fails to highlight the important goal of this study which was to identify evidence-based recommendations. It is highly recommended to focus the title and align it with the study's objective.

**Author Response:** We agree with this comment and will make the appropriate change.

**Change to manuscript:** Title has been changed to:

*“An Evidence-Based Approach to Artificial Intelligence Education for Medical Students: A Systematic Review”.*

**2. Reviewer Comment:** The methodology is well explained and executed. My only concern is the exclusion of the randomized control trial study design from the review. The rationale and decision to not include RCTs must be clearly explained in the methodology.

**Author Response:** We did not exclude randomized controlled trials from the study design. There were no randomized controlled trials published within the scope of our search. We have made this more explicit in the text.

**Change to manuscript:** In the methods section, we have added *“randomized controlled trials (RCTs)”* to the inclusion criteria.

In the results section, we have added *“No RCTs were published within our search criteria”*.

**3. Reviewer Comment:** For example, interventional studies that have looked at the efficacy of utilizing AI for instruction would provide further evidence to contextualize the authors' recommendations and identify more nuanced strengths and weaknesses.

**Author Response:** Respectfully, this query is beyond the scope of our aim and would be more suited to a meta-analysis. We focused on the education of AI concepts and not the use of AI tools for education. The two concepts are closely related, but we limited scope to the former.

**4. Reviewer Comment:** Overall, this is an insightful study that identifies key themes to be addressed in an AI-aware medical curriculum.

**Author Response:** No changes required to be addressed.

**Reviewer #2 (Remarks to the Author):**

**1. Reviewer Comment:** This paper aimed to identify evidence-based recommendations for developing and implementing an AI curriculum in undergraduate medical education through reviewing articles. The authors claimed six themes for the successful implementation of AI in medical school curricula: ethics, theory and application, communication, collaboration, quality improvement, and perception and attitude. Furthermore, they suggested future research about adding formal AI education into medical curricula.

This paper is very timely and novel. The AI education issue is exciting to global medical educators because of ChagGPT. However, the article needs rigorous efforts for good original content and a valid research methodology.

**Author Response:** These comments have been addressed directly in subsequent comments.

**2. Reviewer Comment:** The authors needed to present research gaps from previous review literature sufficiently. This paper is a systematic review, so the authors should show how many review articles have already existed and which research agenda has been explored in these previous review articles. When the authors present current review aims and methodologies, readers can understand the context of the previous literature and the necessity of this research.

**Author Response:** Respectfully, we believe that we have expressed how many review articles related to our research question exist in the first paragraph of the results section. We have broken it down further to make it easier to understand. In addition, we have also included a ROBIS assessment for the review articles as per another suggestion. There are also no concrete research agendas targeting UGME presented by any other studies, only fragments that we used to conduct a thematic analysis and identify key themes for designing and implementing an AI curriculum.

**Change to manuscript:** We have added the following to the Overview section in Methods to further clarify the methodology used:

*“This systematic review also used the Newcastle-Ottawa scale (NOS) and the Risk of Bias in Systematic Reviews (ROBIS) tools to assess bias.^15,16^”*

We have also added the following to the first paragraph in the results section to clarify the number of review articles:

*“Of the 34 total included studies, 23 were evaluated with the NOS criteria and 11 with the ROBIS criteria. One study met the NOS criteria for good^17^ and 22 studies met the NOS criteria for fair.^7,8,18-36,45^ Seven studies met the ROBIS criteria for low risk,^12,13,37,39,40,43,46^ one study was of unclear risk,^38^ and three studies were of high risk.^41,42,44^”*

**3. Reviewer Comment:** The data and analyses did not fully support the authors’ claims that the themes are evidence-based. First, the themes were mainly from review articles, but the authors did not evaluate their quality and evidence level. Instead, they only assessed the quality of the cohort and cross-sectional studies based on the Newcastle-Ottawa quality assessment scale.

**Author Response:** We do agree that the review studies need to be formally assessed. Respectfully, considering the current lack of research within the domain of AI education in UGME, we believe that our data and analyses, with the added evaluation of review studies, support our thematic analysis being evidence-based.

**Change to manuscript:** We have appropriately evaluated the review studies using the ROBIS tool and added the following to the first paragraph of the results section where we discuss risk of bias:

*“Of the 34 total included studies, 23 were evaluated with the NOS criteria and 11 with the ROBIS criteria. One study met the NOS criteria for good^17^ and 22 studies met the NOS criteria for fair.^7,8,18-36,45^ Seven studies met the ROBIS criteria for low risk,^12,13,37,39,40,43,46^ one study was of unclear risk,^38^ and three studies were of high risk.^41,42,44^”*

**4. Reviewer Comment:** Second, the purpose of the previous article did not aim to implement a successful program. Therefore, I don’t think the authors thematized the findings from the included reports. Instead, I guess they thematized the discussion contents from the previous articles. I don't think the discussion contents can be evidence-based.

**Author Response:** As per the methodology, themes were taken from the results of all articles included in this study. We have clarified this in our manuscript as follows:

**Change to manuscript:** In the results section, we have added *“A thematic analysis of the included studies revealed six themes: ethics, theory and application, communication, teamwork, quality improvement, and perception and attitude”*.

**5. Reviewer Comment:** Therefore, I suggest the authors collect and evaluate articles implementing and evaluating AI programs to support the claims that recommendations are evidence-based. In addition, they should analyze the outcomes of articles related to the research questions.

**Author Response:** Respectfully, there are no articles discussing/evaluating the implementation of an AI education program or evaluation of an implemented AI education program in the undergraduate medical curricula. We are collecting all the evidence we have related to AI education and compiling it to serve as a backbone for a future paper where we aim to create an AI education curriculum for undergraduate medical students. This will be a novel achievement, so we cannot base it off of pre-existing work.

**6. Reviewer Comment:** Additional work to improve the paper. Rather than a systematic review, this paper seems more likely to be an integrative or scoping review. The authors wanted to explore implementation strategies using thematic analysis. This aim matches well with the integrative review.

**Author Response:** Respectfully, we believe that this paper fits the definition of a systematic review rather than a scoping or integrative review. As per Munn et al. (2018), “conducting a systematic review may also identify gaps, deficiencies, and trends in the current evidence and can help underpin and inform future research in the area”. Munn et al. (2018), also identify the following five indications for a systematic review: “(1) uncover the international evidence; (2) confirm current practice/address any variation/identify new practices; (3) identify and inform areas for future research; (4) identify and investigate conflicting results; and (5) produce statements to guide decision-making”. As our goal was “to identify and aggregate the current evidence-based recommendations for the development and implementation of an AI curriculum in undergraduate medicine”, this satisfies all five indications for a systematic review. In addition, we performed a risk of bias review of all included studies, which is not necessary for the other types of reviews.

Munn Z, Peters MD, Stern C, Tufanaru C, McArthur A, Aromataris E. Systematic review or scoping review? Guidance for authors when choosing between a systematic or scoping review approach. BMC medical research methodology. 2018; 18:1-7.

**7. Reviewer Comment: Or the authors could narrow the focus on survey or review articles to improve the research design.**

**Author Response:** As there is already a lack of literature surrounding this topic, we believe that further limiting ourselves to only survey or review articles would result in us missing other important information.

**8. Reviewer Comment:** There are three more things. One is that the results used non-included articles from 50 to 65. I think the authors should remove them.

**Author Response:** We acknowledge that those studies should be removed. We have reviewed and confirmed that the studies were correctly excluded from our initial review.

**Change to manuscript:** In the results section, we have removed all references and links to the non-included articles and have changed the text to only reflect the included articles.

*“Regulatory challenges include integrating AI into clinical settings and workflow, data sharing between institutions, and the translation and interpretation of AI models in a health- care setting.^3,13,38^ From an economic perspective, learning about the various economic considerations with respect to AI is crucial to having an understanding of the business and clinical aspects of AI implementation.^13^ From an organizational culture perspective, including legal strategies and governance in programs can build awareness regarding the ethics, inclusion, equity, patent rights, and confidentiality when using AI tools.^3,5,13^”*

**9.** **Reviewer Comment:** Another is the discussion. AI in healthcare seems unrelated to the research questions in this paper. I hope to see more detailed and profound talks about developing and implementing AI education from the results.

**Author Response:** We acknowledge that AI in healthcare may seem beyond the scope of the research question; however, the goal of introducing AI education in UGME curricula is to prepare future physicians for the integration of AI in healthcare. We have made this more clear in the text.

**Change to manuscript:** When discussing AI in healthcare we reference the development of necessary skills learned in UGME.

In the third paragraph of the results, we changed:

*“In terms of the implications of implementation, developing the ability to visualize how the role and workflow of a physician may change with AI during UGME,^13,33^ and learning implementation techniques that would help with the AI transition, may improve the future integration of AI in healthcare.”*

In the fifth paragraph of the discussion, we changed:

*“AI-interested physicians can also focus on implementation communication, using the skills they developed during UGME and elsewhere to better connect AI with healthcare.”*

**10. Reviewer Comment:** The other is Figure 2. As a reader, I would like to see the quality level in Table 1 instead of Figure 2.

**Author Response:** We agree that a table would increase clarity and be beneficial for readers.

**Change to manuscript:** We have added two columns to Table 1 to report the overall NOS and ROBIS scores. Furthermore, we have added Tables 2 and 3 to appendix A to breakdown the individual risk of bias scores for each study.

| Title | Selection | Comparability | Outcome (Cohort, Cross-sectional) |
| --- | --- | --- | --- |
| The impact of artificial intelligence on clinical education: perceptions of postgraduate trainee doctors in London (UK) and recommendations for trainers^7^ | 3 | 1 | 1 |
| Health Care Students’ Perspectives on Artificial Intelligence: Countrywide Survey in Canada^8^ | 3 | 1 | 1 |
| Medical artificial intelligence readiness scale for medical students (MAIRS-MS) – development, validity and reliability study^17^ | 3 | 2 | 1 |
| Influence of Artificial Intelligence on  Canadian Medical Students'  Preference for Radiology Specialty: A  National Survey Study^18^ | 3 | 1 | 1 |
| Medical students' attitude towards artificial intelligence:  a multicentre survey^19^ | 2 | 1 | 1 |
| Attitudes and perceptions of UK medical students towards artificial intelligence and radiology: a multicentre survey^20^ | 3 | 1 | 2 |
| Medical Student Perspectives on the Impact of Artificial Intelligence  on the Practice of Medicine^21^ | 3 | 1 | 1 |
| Impact of artificial intelligence on US medical students' choice of radiology^22^ | 3 | 1 | 2 |
| Radiology Community Attitude in Saudi Arabia about the  Applications of Artificial Intelligence in Radiology^23^ | 2 | 1 | 2 |
| Interventional radiology and artificial  intelligence in radiology: Is it time to enhance the vision of our medical students?^24^ | 3 | 1 | 1 |
| The Approaches and Expectations of the Health Sciences Students Towards Artificial Intelligence^25^ | 2 | 1 | 1 |
| Artificial intelligence and medical education: A global mixed-methods study of medical students’ perspectives^26^ | 3 | 1 | 1 |
| Medical Students’ Perceptions towards Digitization and Artificial Intelligence: A Mixed-Methods Study^27^ | 3 | 1 | 1 |
| Machine learning in medical education: a survey of the experiences and opinions of medical students in Ireland^28^ | 2 | 1 | 1 |
| A survey on the future of radiology among radiologists, medical students  and surgeons: Students and surgeons tend to be more skeptical about artificial intelligence and radiologists may fear that other disciplines take over^29^ | 3 | 1 | 2 |
| Are We Ready to Integrate Artificial Intelligence Literacy into Medical School Curriculum: Students and Faculty Survey^30^ | 3 | 1 | 1 |
| Artificial Intelligence and the Future of Primary Care: Exploratory Qualitative Study of UK General Practitioners’ Views^31^ | 3 | 1 | 1 |
| Computerization and the future of primary care: A survey of general practitioners in the UK^32^ | 3 | 1 | 1 |
| US primary care in 2029: A Delphi survey on the impact of machine learning^33^ | 3 | 0 | 1 |
| Medical student knowledge and critical appraisal of machine  learning: a multicentre international cross-sectional study^34^ | 3 | 1 | 1 |
| Differences in Knowledge and Perspectives on the Usage of Artificial Intelligence Among Doctors and Medical Students of a Developing Country: A Cross-Sectional Study^35^ | 2 | 2 | 2 |
| Mapping the Access of Future Doctors to Health Information Technologies Training in the European Union: Cross-Sectional Descriptive Study^36^ | 2 | 1 | 2 |
| Artificial Intelligence in Primary Health Care: Perceptions, Issues, and Challenges^45^ | 2 | 2 | 1 |

| **Review** | **Phase 2** | | | | **Phase 3** |
| --- | --- | --- | --- | --- | --- |
|  | 1.Study Eligibility | 2.Identification and selection of studies | 3.Data collection and study appraisal | 4.Synthesis and findings | Risk of bias in review |
| Artificial Intelligence in Undergraduate Medical Education: A Scoping Review^12^ | Low Risk | Low Risk | High Risk | Low Risk | Low Risk |
| Artificial Intelligence Education Programs for Health Care Professionals: Scoping Review^13^ | Low Risk | Low Risk | Unclear | Low Risk | Low Risk |
| Stakeholders’ perspectives on the future of artificial intelligence  in radiology: a scoping review^37^ | Low Risk | Low Risk | Unclear | Low Risk | Low Risk |
| Applications and Challenges of Implementing Artificial Intelligence in Medical Education: Integrative Review^38^ | Low Risk | High Risk | High Risk | Low Risk | Unclear |
| Artificial Intelligence Education and Tools for Medical and Health Informatics Students: Systematic Review^39^ | Low Risk | Low Risk | Unclear | Low Risk | Low Risk |
| Medical education trends for future physicians in the era of advanced technology and artificial intelligence: an integrative review^40^ | Low Risk | Low Risk | Low Risk | Low Risk | Low Risk |
| The Recent Progress and Applications of Digital Technologies in Healthcare: A Review^41^ | High Risk | High Risk | High Risk | High Risk | High Risk |
| Digital health competencies in medical school education: a scoping review and Delphi method^42^ | High Risk | High Risk | High Risk | Low Risk | High Risk |
| Digital Health Training Programs for Medical Students: Scoping Review^43^ | Low Risk | Low Risk | Unclear | Low Risk | Low Risk |
| Systematic Review of Radiologist and Medical Student Attitudes on the Role and Impact of AI in Radiology^44^ | Low Risk | High Risk | High Risk | High Risk | High Risk |
| Educating Future Physicians in Artificial Intelligence (AI): An Integrative Review and Proposed Changes^46^ | Low Risk | Low Risk | High Risk | Low Risk | Low Risk |

**11. Reviewer Comment:** The authors did not present a registered protocol. So, it would be good for the authors to register the research protocol before starting the study.

**Author Response:** We acknowledge that this should have been registered before, but as the study is completed, we cannot make a change.

**12. Reviewer Comment:** This paper needs to describe a more detailed methodology for reproduction. The authors tried to follow the PRISMA guideline, but more was needed. PRISMA 2020 for abstracts and AMSTAR 2 checklist will be helpful.

**Author Response:** Respectfully, we believe that the PRISMA guideline was adequately followed during this study. We also do agree that using the PRISMA 2020 for abstracts would be helpful and it was incorporated as per another comment.

**13.** **Reviewer Comment:** First, the abstract should include more detailed information, such as specific inclusion and exclusion criteria, information sources, methods to assess the risk of biases in studies, and limitations of the evidence.

**Author Response:** As per the PRISMA 2020 for abstracts checklist, we agree with these suggestions and have made the necessary changes.

**Change to manuscript:** In the abstract, we added information about the inclusion and exclusion criteria, information sources, methods of assessing risk of bias, and limitations of evidence:

*“Six databases were searched from inception to April 1 2022 for cross sectional and cohort studies of fair quality or higher on the Newcastle-Ottawa scale, systematic, scoping, and integrative reviews, randomized controlled trials, and Delphi studies about AI education in undergraduate medical programs. The search yielded 991 results, of which 27 met all the criteria and seven more were included using reference mining.”*

*“Despite the limitations of a high degree of heterogeneity among the study types and a lack of follow-up studies evaluating the impacts of current AI strategies, a thematic analysis of the key AI principles identified six themes needed for a successful implementation of AI in medical school curricula.”*

**14. Reviewer Comment:** Second, key research questions did not specify PICO and others, such as time, setting, or study design.

**Author Response:** Respectfully, we believe that the PICO model does not apply to our study as we have no comparator. We will make the population and outcomes of our study more clear.

**Change to manuscript:** In the last paragraph of the introduction, we changed and added:

*“The purpose of this systematic review is to identify and aggregate the current evidence-based recommendations for the development and implementation of an AI curriculum in undergraduate medical education (UGME). We hope that exposing medical students to an AI curriculum in the future will result in an increased integration of AI into their future practice and improved competency when using AI in medicine.”*

**15. Reviewer Comment:** Third, inclusion criteria only described article types, with no exclusion criteria. I wanted to know whether the authors include non-English articles, experimental studies, or single-institution studies. Besides, I asked whether the authors excluded low-quality articles.

**Author Response:** Respectfully, the exclusion criteria are stated in the section under Assessment of study eligibility. The exclusion criteria include: non-English studies, post-graduate medical education, papers targeting allied healthcare workers, original research that focuses on applications of AI and not AI education, conference abstracts, case reports, narrative studies, studies assessed as poor quality using the NOS. We believe that this exclusion criteria answers the above comment.

**16. Reviewer Comment:** Fourth, two or more researchers should select articles independently. The authors described two researchers conducting study screening, data extraction, and thematic analysis. However, they should have commented on how many researchers perform study selection and quality assessment.

**Author Response:** We agree that more clarity is needed regarding how many researchers performed study selection and quality assessment. We have added information that addresses this.

**Change to manuscript:** In the quality assessment and statistical analysis section, we have added a statement outlining the number of researchers that performed the quality assessment.

*“Quality assessment was performed by two reviewers (NP and AG) using the NOS criteria”*.

**17.** **Reviewer Comment:** Fifth, I wondered why the authors chose NOS because NOS can evaluate only cohort and case-control studies. Many other evaluation tools exist, such as AMSTAR, MERSQI, or BEME. This paper included various articles, so I suggest the authors use more appropriate tools for surveys and review articles.

**Author Response:** We agree that more than one evaluation tool is needed and we have addressed this in another comment. We selected to use the ROBIS tool to evaluate review studies as it is an accredited tool. The changes associated with the ROBIS tool have been addressed in another comment.

In terms of why choosing the NOS specifically, Zeng et al. (2020) indicated that the NOS tool is the most commonly used tool for cohort and case-control studies. Furthermore, Cook and Reed (2015), compared the NOS and MERSQI, concluding that they are both reliable tools that correlate well.

Ma, LL., Wang, YY., Yang, ZH. et al. Methodological quality (risk of bias) assessment tools for primary and secondary medical studies: what are they and which is better?. Military Med Res 2020; 7: 1-11.

Cook, DA., Reed, DA. Appraising the Quality of Medical Education Research Methods: The Medical Education Research Study Quality Instrument and the Newcastle–Ottawa Scale-Education. Acad Med 2015; 90: 1067-1076.

**18. Reviewer Comment: The manuscript was understandable, but needed to check for omissions and errors.**

**Author Response:** We interpret this comment as an overall summarizing comment that will be addressed by our other changes.

**19. Reviewer Comment: In addition, for readers who are not familiar with article evaluation tools, NOS only is difficult to understand.**

**Author Response:** We agree that readers may not be familiar with the NOS criteria, so we will make a change to clarify the NOS.

**Change to manuscript:** In the quality assessment and statistical analysis section, we have added a description of the NOS to allow readers to become better acquainted with the NOS criteria.

*“The NOS evaluates studies based on the selection of the study groups, the comparability of groups, and the ascertainment of either the exposure or outcome of interest.^15^”*

**20. Reviewer Comment:** Furthermore, the authors said that they separated the previous studies into survey and interventional. Interventional studies were mainly reports and reviews, which confused me because interventional studies usually mean experimental studies. Therefore, I suggest the authors change the term ‘interventional’ and explain the definition of it.

**Author Response:** We agree with this comment and will be changing the groups we divide the studies into.

**Change to manuscript:** All instances of the term “interventional studies” have been replaced with “non-survey studies” to account for the diversity in types of studies in this category.

**Reviewer #3 (Remarks to the Author):**

**1. Reviewer Comment:** Pupic et al. did a systematic review on the AI-specific education in medical schools. They had collected more than 900 original paper and scoped down into 30 ones fit the study criteria. In conclusion they found six core themes should be suggested implant into the future AI related medical education.

**Author Response:** We interpret this to be a summarizing comment that requires no changes to the manuscript.

**2. Reviewer Comment:** It is also mentioned some field of medical education developed more dependent on current state of art technique of AI, while other filed less developed. So, it is suggested to draw a bar chart to demonstrate the diversity of the current AI application situation in various medial field or specialty. And the distribution of the 30 selected paper should also be more elucidated on each research field.

**Author Response:** Respectfully, we believe that this is outside the scope of our study as we are focusing on what students should be taught so they can apply their AI knowledge to any medical specialty.

**3. Reviewer Comment:** The specific indicators of quality assessment and statistical analysis should be clearer. The current presentation format of Table 1 and Figure 2 and 3 are not strait forward and difficult to link with the result part.

**Author Response:** Respectfully, we believe that Table 1 is a descriptive table of the included studies that is presented in this format in many systematic reviews. We also believe that Figure 3 visually depicts how many studies support each theme in a simple manner. We do agree that Figure 2 is difficult to interpret and will change it into a table in agreement with another comment.

**Change to manuscript:** In the results section, we have changed Figure 2 into Table 2 which breaks down the individual NOS scoring for all relevant studies to better outline the risk of bias assessment. We have also added a Table 3 for individual ROBIS scoring. Both of these tables were added into Appendix A. We have added the overall NOS and ROBIS scores to Table 1 as one column.This is seen above in the response to comment 10 from reviewer 2.

**4. Review Comment:** In all, the topic is meaningful and the research method was thoroughly with high efficiency and plentiful of data. The current manuscript should be accepted after the minor revisions.

**Author Response:** We interpret this to be a summarizing comment that requires no changes to the manuscript.
